# Development of a machine learning-derived model to predict unplanned ICU admissions after major non-cardiac surgery

**DOI:** 10.1186/s12871-025-03195-8

**Published:** 2025-07-17

**Authors:** Catherine Chiu, Matthias R. Braehler, Anne L. Donovan, Atul J. Butte, Romain Pirracchio, Andrew M. Bishara

**Affiliations:** 1 Department of Anesthesia and Perioperative Care, University of California, San Francisco, USA; 2 Bakar Computational Health Sciences Institute, University of California San Francisco, 550 16th St. Office 5212, San Francisco, CA 94158 USA

**Keywords:** Unanticipated ICU admission, Machine learning, Artificial intelligence, Predictive analytics

## Abstract

**Background:**

Unplanned postoperative intensive care unit admissions (UIAs) are rare events that cause significant challenges to perioperative workflow. We describe the development of a machine-learning derived model to predict UIAs using only widely used preoperative variables.

**Methods:**

This was a 3-year retrospective review of all adult surgeries under the General, Vascular, and Thoracic surgical services with anticipated length of greater than 180 minutes at a single institution. A UIA was defined as any post-operative patient recovering in the post-anesthesia care unit (PACU) requiring direct transfer to the intensive care unit (ICU) for higher level of care. We developed our prediction model with a gradient-boosting decision tree algorithm (XGBoost). The model incorporated sixteen generalizable predictor variables that were derived from the demographics and surgical booking details. Validation and evaluation were performed with 10-fold cross validation, and model performance was evaluated using the area under the receiver operating characteristic (ROC) curve, sensitivity, specificity, and likelihood ratio.

**Results:**

A total of 4658 patients were included for analysis. The incidence of UIAs was 2.3%. With 10-fold cross validation, the area under the ROC curve was 0.80 (95% CI 0.74–0.86). Two decision thresholds were used, which achieved the best specificity of 94% (95% CI 92–96%), best positive likelihood ratio of 4.22 (95% CI 0.99–8.79), and best sensitivity of 82% (95% CI 58–100%).

**Conclusions:**

Our machine learning-derived model is a reliable tool for the perioperative clinician to predict a rare outcome in high-risk patients using only preoperative variables. Future studies will include prospective validation of this model at other institutions and real-time incorporation for improvement in perioperative workflow.

**Supplementary Information:**

The online version contains supplementary material available at 10.1186/s12871-025-03195-8.

## Introduction

Unplanned postoperative intensive care unit (ICU) admissions (UIAs) are rare but significant events for patients, nurses, surgical and anesthesia teams, and the hospital system [1–4], with a reported incidence varying widely from 0.12% up to 6.7%.It is well described that UIAs are associated with increased morbidity and mortality, likely due to the de-facto requirement for ICU-level care such as need for vasopressors, transfusions, or mechanical ventilation [1, 2, 5, 6]. 

From the perspective of perioperative workflow, UIAs lead to higher strain on the post anesthesia care unit (PACU), including higher intensity nursing needs and longer recovery time in the PACU [7]. These strains in turn can lead to an operating room backlog if patients cannot immediately be moved from the operating room to the PACU [8]. Furthermore, immediate transfer from the PACU to ICU results in multiple patient-care handovers that increases the probability of communication errors [9]. Beyond workflow disruptions, unplanned ICU transfers incur markedly higher hospital costs and poorer patient-reported outcomes than planned critical-care admissions, underscoring the value of accurate pre-operative triage [10]. 

Prediction models have been developed for earlier identification of prolonged PACU recovery times and postoperative ICU needs [11–13]. One group developed several models predicting UIAs using traditional regression; however, the most accurate model required use of intraoperative data [11]. The limited performance of prediction models that are based on traditional parametric algorithms is likely due to the unrealistic expectations for population datasets, including assumptions of normalcy, homogeneity of variance, and absence of multicollinearity [14]. As mentioned in Gabriel et al., although a prediction model that includes intraoperative data is important for guidance in patient care, such a model is less impactful in improving perioperative workflow efficiency [12]. A more useful model would only incorporate preoperative variables such that prediction could occur prior to the day of surgery to facilitate appropriate allocation of increasingly important ICU beds.

We hypothesize that machine learning algorithms offer superiority over traditional parametric approaches, especially in the prediction of rare outcomes such as UIAs. In this retrospective study at a single institution, we developed and retrospectively validated a classification model using a tree-based boosting algorithm to determine unplanned postoperative ICU admissions in patients undergoing major non-cardiac surgery upon discharge from the PACU with generalizable variables that are readily available prior to the day of surgery. Finally, we demonstrate how to interpret our machine-learning derived model to help guide decision-making around ICU bed allocation.

## Methods

Approval for a retrospective review of electronic medical records was obtained by the University of California, San Francisco (UCSF) Institutional Review Board (IRB, # 19–27979), and the requirement for written informed consent was waived by the IRB. This study included all patients over the age of 18 who underwent surgery under the General Surgery, Vascular Surgery, and Thoracic Surgery services at UCSF, a non-trauma, non-obstetric, tertiary care center between December 2016 and December 2019. We limited our analysis to cases with anticipated durations greater than 180 min, as UIA rose from 0.79% in surgeries < 3 h to 2.25% in those ≥ 3 h—a pattern corroborated by prior studies [15]—and this cutoff optimizes both sample size and event rate for our ML model. The outcome of an unexpected ICU admission was defined as any postoperative patient admitted to the PACU for recovery, as determined by a bed location marker, and who was directly transferred to an ICU destination upon discharge from the PACU for persistent requirement of intensive care. We used Strengthening the Reporting of Observational Studies in Epidemiology (STROBE) guidelines and Transparent Reporting of a Multivariable Prediction Model for Individual Prognosis or Diagnosis (TRIPOD) guidelines throughout this investigation for predictive model development and validation.

### Data collection and pre-processing

We extracted patient records from our institution’s EHR system clinical data warehouse (Epic-Clarity). Many predictor variables (> 800 after one-hot-encoding and removal of rarely occurring values) were chosen (Supplemental Table 1) for their likelihood to predict the outcome and based on positive associations from prior publications [2, 6, 11]. Embeddings defined from diagnosis codes allowed us to improve the performance by finding nuanced interactions between preoperative diagnoses and the operation being performed. These ICD10 codes were converted to strings, then to Word2vec vectors, and then to embeddings by calculating the mean of the vector for each string. We also examined the AWOL delirium-prevention score—an easily calculated tool incorporating Age > 80, ability to spell ‘World’ backwards, Orientation to hospital location (city, state, hospital name and floor), and a nursing illness‐severity rating—as one of our preoperative predictors [16]. For data manipulation and analysis, we employed a Python-based computational framework incorporating several specialized libraries for data science and machine learning applications, including an implementation of gradient boosted decision trees. In addition to embeddings, every ICD-10 diagnosis was matched to its corresponding ICD-10 category (21 available), and the total sum of diagnoses per category were used as features. Numeric outliers greater than three standard deviations from the mean were detected, and these patients were removed from the dataset due to concern for errors in data entry. Missing data for numerical data were not manipulated and for categorical data were labeled as ‘missing’. After data preprocessing, the entire dataset was randomly split into a training, validation, and test datasets, 56%−14%−30% respectively, for model development. Positive cases in the training set were up sampled to 50% of the total cases in the training set to improve the prediction of the rare outcome. The test dataset was left untouched throughout model development. Following complete model development, we applied preprocessing transformations from the training set to the test dataset variables.

### Statistical analysis

Descriptive statistics were performed using Python. We compared parametric numeric variables using t-tests to assess differences in means and standard deviations, while non-parametric numeric variables were analyzed with the Kruskal-Wallis test to evaluate differences in medians and interquartile ranges. For categorical variables, variables with less than five occurrences were tested with Fisher’s exact test and chi-square for all other variables.

### Data classification and performance evaluation

Our predictive approach employed a tree-based ensemble learning framework (XGBoost) that progressively enhances prediction accuracy through sequential model refinement [17]. We chose this methodology for its computational efficiency, robust performance with imbalanced datasets, and native capacity to process incomplete data entries without requiring imputation.

Model parameter optimization was conducted using the validation dataset, adjusting key algorithm settings (including learning rate of 0.01, tree depth maximum of 4, child weight minimum of 2, 1000 estimators, and positive weight scaling of 1). The optimized model was subsequently evaluated on the separate test dataset to determine discrimination capability, along with sensitivity and specificity metrics determined through bootstrapping. We determined two decision thresholds from the validation dataset for clinical use: a low threshold at 5% designed to maximize sensitivity and a high threshold at 30% to maximize specificity of UIA (Supplemental Fig. 1). We additionally calculated both positive and negative likelihood ratios based on our institution’s prevalence, and the likelihood ratio nomograms were used to plot pre-test and post-test probabilities.

### Model visualization

SHapley Additive exPlanations (SHAP) [18, 19], an explainable AI tool based in game theory, was used to interpret our model. This technique converts the complex decision-making of the model into accessible visualizations that show how individual variables contribute to predictions. The visualizations summarize the intensity and directionality of each predictor’s effect on the model’s risk assessment (Fig. 3). This SHAP package can also produce a decision plot to explain the decision in a specific case (Fig. 4).

## Results

**Fig. 1:**
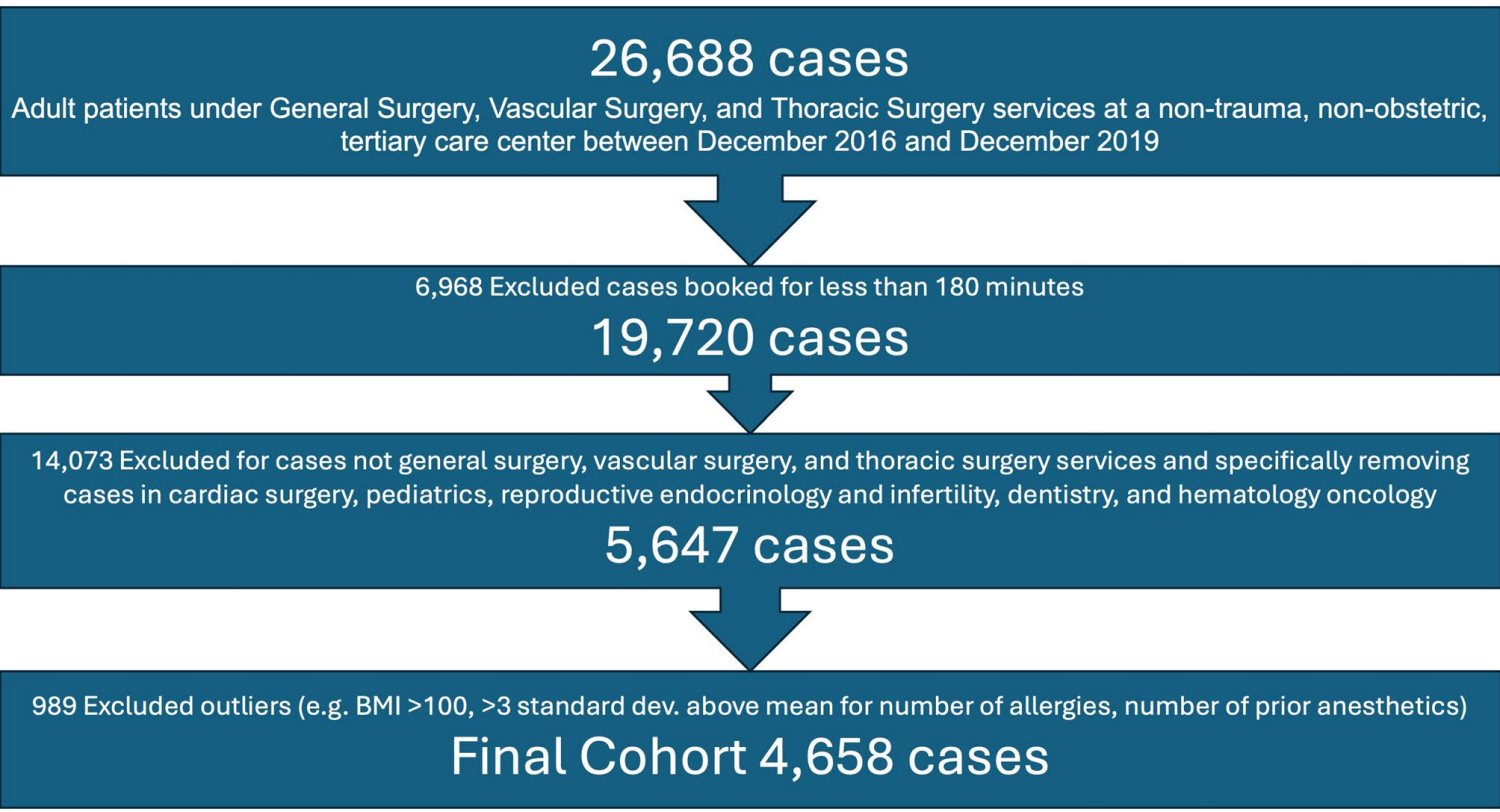
Shows the inclusion flow diagram of how patients were included in the cohort of this study

Out of 26,688 patients who underwent any type of surgery during this time, a total of 4658 patients were included in analysis (Fig. 1). Of the final dataset, 105 patients (2.3%) had an unplanned postoperative ICU admission. These 4658 patients were split into the following groups: training dataset (2608 patients), validation dataset (652 patients), and test dataset (1398 patients). Patients who experienced a UIA tended to be older, male, to have a higher ASA class (3–4), and longer length of stay after surgery (Table 1). These patients tended to have a higher absolute number of past medical diagnoses by ICD-10 codes in the categories of Circulatory and Digestive systems. In terms of surgical characteristics, there were differences in UIAs based on cases classified as emergent (Table 2). Procedures that contained the following text at time of booking were less likely to result in a UIA: cholecystectomy, colectomy, gastric, hernia, laparoscopic, ‘-ostomy’, resection, and robotic. Procedures that contained the following text at time of booking were more likely to result in a UIA: aneurysm, bronchoscopy, carotid, open, stent, thoracic, and thoracotomy.

**Table 1:** Patient demographics

	non-UIA (*n* = 4553)	UIA (*n* = 105)	*p*-value
Age (mean (SD))	59.4 (15.3)	64.1 (12.7)	< 0.001*
Gender = Male (%)	2175 (47.8)	61 (58.1)	0.046^†^
Body Mass Index (kg/m^2^) (mean (SD))	28.4 (7.7)	29.4 (7.3)	0.159*
ASA Class (%)			< 0.001^†^
1	103 (2.3)	1 (1.0)	
2	2159 (47.4)	20 (19.0)	
3	2152 (47.3)	69 (65.7)	
4	119 (2.6)	15 (14.3)	
5	1 (0.0)		
Missing	19 (0.4)		
Prior admission in the past 30 days (%)	2320 (51.0)	48 (45.7)	0.335^†^
Prior admission in the past 90 days (%)	3428 (75.3)	72 (68.6)	0.144^†^
Number of anesthetic encounters in the previous 3 years (mean (SD))	0.9 (1.5)	1.1 (1.4)	0.387*
I00-I99 (Diseases of the circulatory system): Mean (SD)	1.1 (1.5)	1.8 (2.0)	0.001*
K99-K93 (Diseases of the digestive system): Mean (SD)	1.1 (1.4)	0.6 (0.9)	< 0.001*
Length of stay in days after surgery: Mean (SD)**	7.3 (8.8)	12.3 (15.1)	0.001

^*^p-value derived from t-test

^†^*p*-value derived from Chi-square test

**30 cases are missing length of stay calculation

**Table 2:** Surgical characteristics

	non-UIA (*n* = 4553)	UIA (*n* = 105)	*p*-value
Emergent case	984 (21.6)	32 (30.5)	0.040^†^
Procedures containing the words/characters: True (%)			
‘Aneurysm	40 (0.9)		1.00^†^
‘Aortic	28 (0.6)		1.00^†^
‘Bronchoscopy	340 (7.5)	26 (24.8)	< 0.001^†^
‘Carotid	59 (1.3)	14 (13.3)	< 0.001^†^
‘Cholecystectomy’	256 (5.6)	1 (1.0)	0.063 ^†^
‘Colectomy	601 (13.2)	1 (1.0)	< 0.001^†^
‘Endo-	30 (0.7)		1.00^†^
‘Gastric	165 (3.6)	0 (0.0)	0.053^†^
‘Graft’	12 (0.3)	1 (1.0)	0.257^‡^
‘Hernia’	525 (11.5)	6 (5.7)	0.089^†^
‘Laparoscopic	2058 (45.2)	15 (14.3)	< 0.001^†^
‘Open’	698 (15.3)	19 (27.6)	< 0.001^†^
‘-Ostomy’	848 (18.6)	6 (5.7)	< 0.001^†^
‘Resection’	734 (16.1)	8 (5.7)	0.006^†^
‘Robotic’	667 (14.6)	7 (6.7)	0.031^†^
‘-Scopy’	389 (8.5)	14 (13.3)	0.121^†^
‘Stent’	197 (4.3)	15 (10.5)	1.00^†^
‘Thoracic’	9 (0.2)		1.00^‡^
‘Thoracotomy’	332 (7.3)	26(24.8)	< 0.001^†^
‘Vasc-‘	30 (0.6)		1.00^†^

^†^*p*-value derived from Chi-square test

^‡^*p*-value derived from Fisher’s exact test

**Fig. 2:**
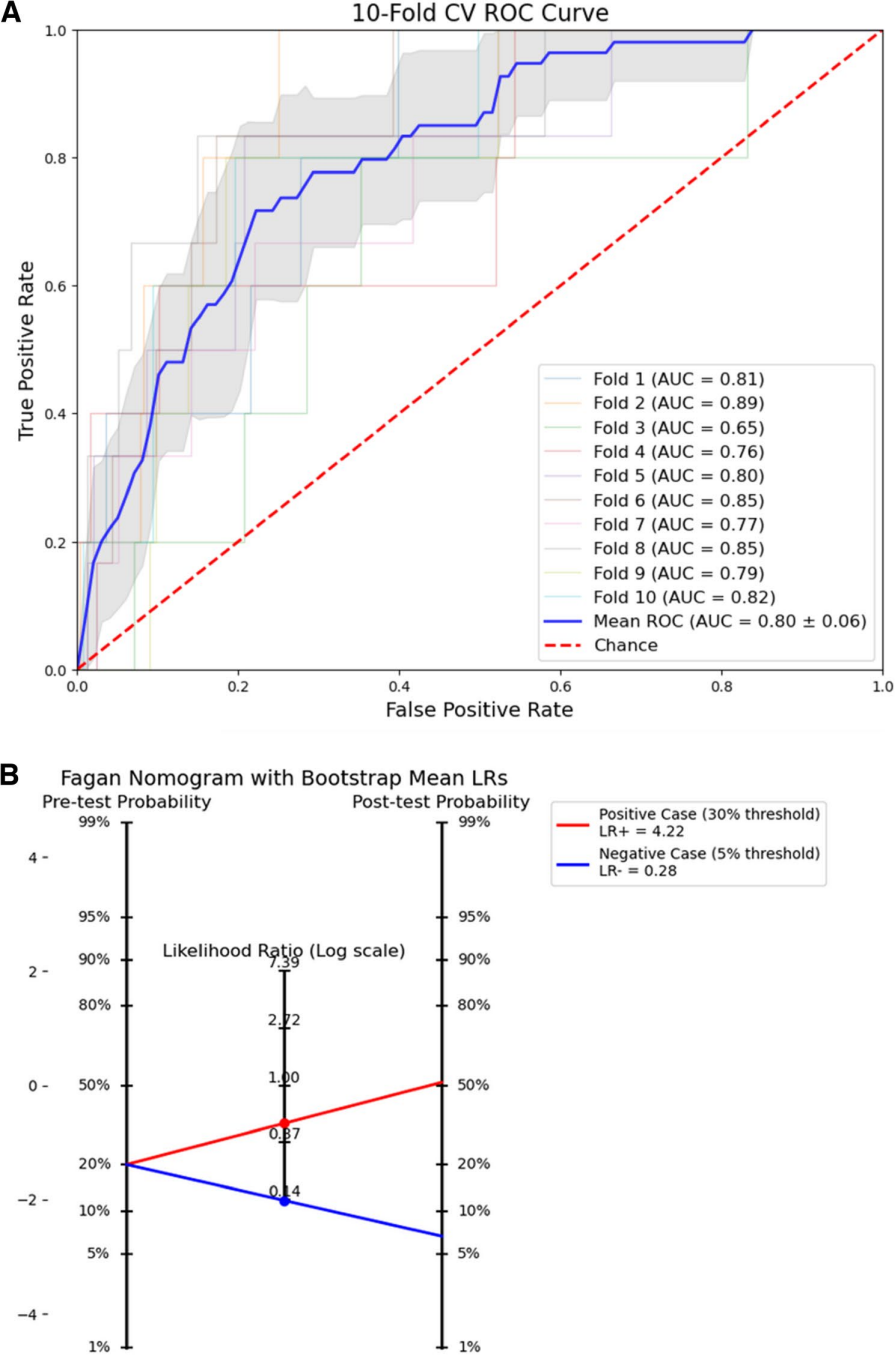
Evaluation of model and likelihood ratios nomograms. **A** Receiver Operator Characteristic (ROC) Curve on test dataset with 10-fold cross validation. **B** Likelihood Ratio Nomograms. Red line: pre- and post-test probability for all patients (prevalence 2.3%). Red and blue lines show how the test provides convergence in post-test probability for a patient with a pre-test probability of 20% chance of needing UIA

The final model was derived from a gradient boosting algorithm as outlined in the Methods section 10-fold cross-validation performance was reported. The final AUC for the ROC curve was 0.80 (95% CI 0.74–0.86) (Fig. 1A). Using the pre-determined lower decision threshold of 5%, our model achieves a mean sensitivity of 81% (95% CI 59–100%), mean specificity of 66% (95% CI 62–70%), mean positive likelihood ratio of 2.37 (95% CI 1.72–2.97), and mean negative likelihood ratio of 0.28 (95% CI 0.00–0.60). Using the pre-determined higher decision threshold of 30%, our model achieves a mean sensitivity of 25% (95% CI 6–47%), mean specificity of 94% (95% CI 92–96%), mean positive likelihood ratio of 4.22 (95% CI 0.99–9), and mean negative likelihood ratio of 0.80 (95% CI 0.56–1.01). A likelihood ratio nomogram was used to determine the post-test probability of UIA with a positive model prediction with the higher, more specific threshold (Fig. 2B).

**Fig. 3:**
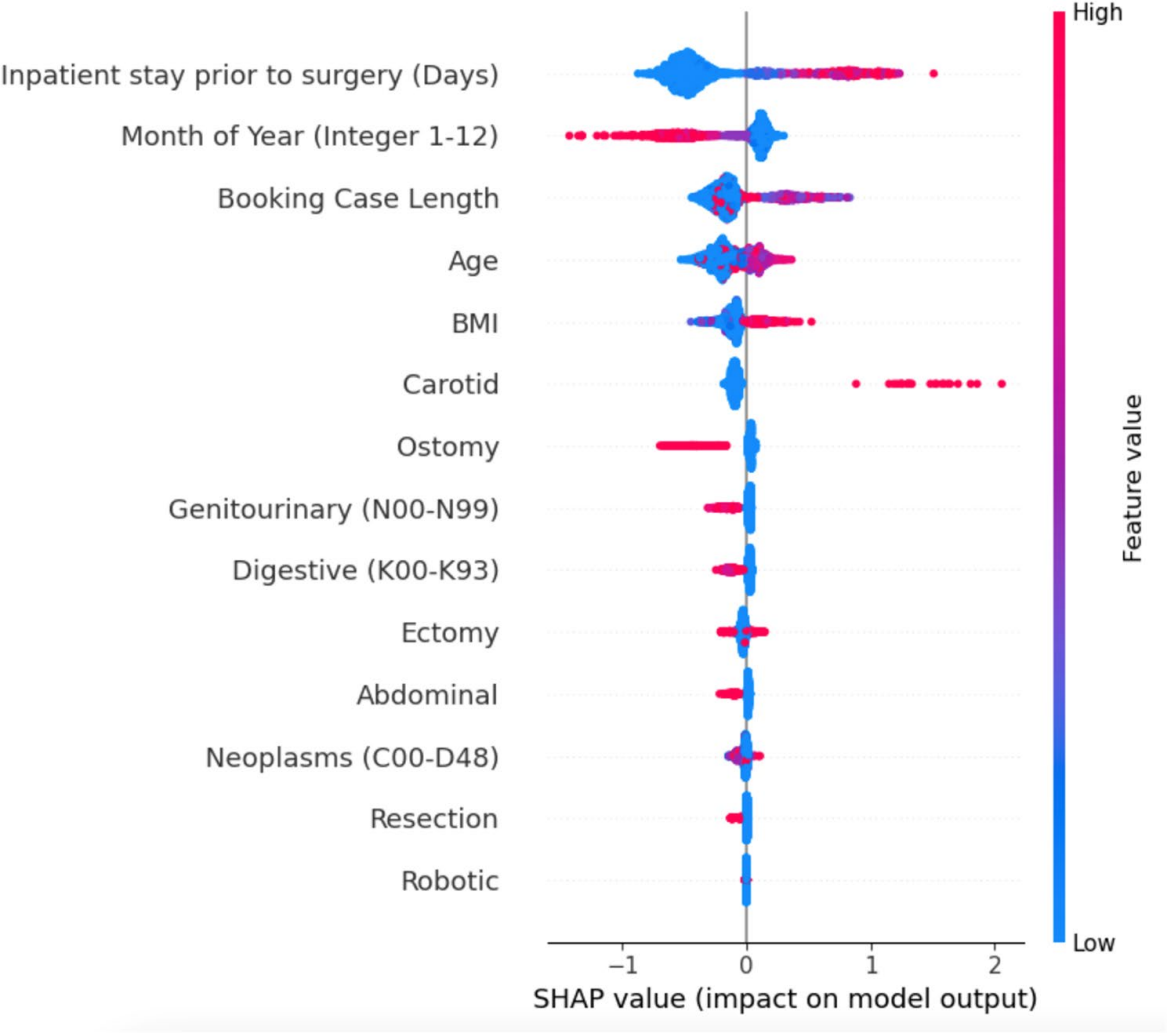
Model summary of the most influential variables. Model summary of the most influential variables. Each dot represents an individual case-variable pair. The x-axis shows the SHAP value (impact on log-odds of UIA): dots to the right increase predicted risk, dots to the left decrease it. Red dots indicate higher numeric value, and blue dots indicate lower numeric value. Dots (regardless of color) on the right side of the y-axis indicate a higher impact value on the model to predict a positive UIA, and vice versa. Dots with the same impact value are plotted on top of each other to create a vertical distribution

Figure 3 displays a SHAP summary plot for the most influential variables (i.e., feature importance) for the final model.

### How to read the SHAP summary plot

In Fig. 3, each point represents one patient’s contribution from a single feature. The horizontal position is the SHAP value—how much that feature pushed the prediction toward (positive) or away from (negative) UIA. Point color reflects the original feature value (red = high; blue = low). For example, red points far to the right mean that high values of that feature strongly increase UIA risk.

Notably, the model was heavily influenced to positively predict a UIA for patients with higher BMI and age undergoing long or carotid-related surgery who had long inpatient stays prior to surgery. The model was heavily influenced to negatively predict a UIA in patients with a history of digestive tract or genitourinary disease or undergoing laparoscopic and robotic procedures.

**Fig. 4:**
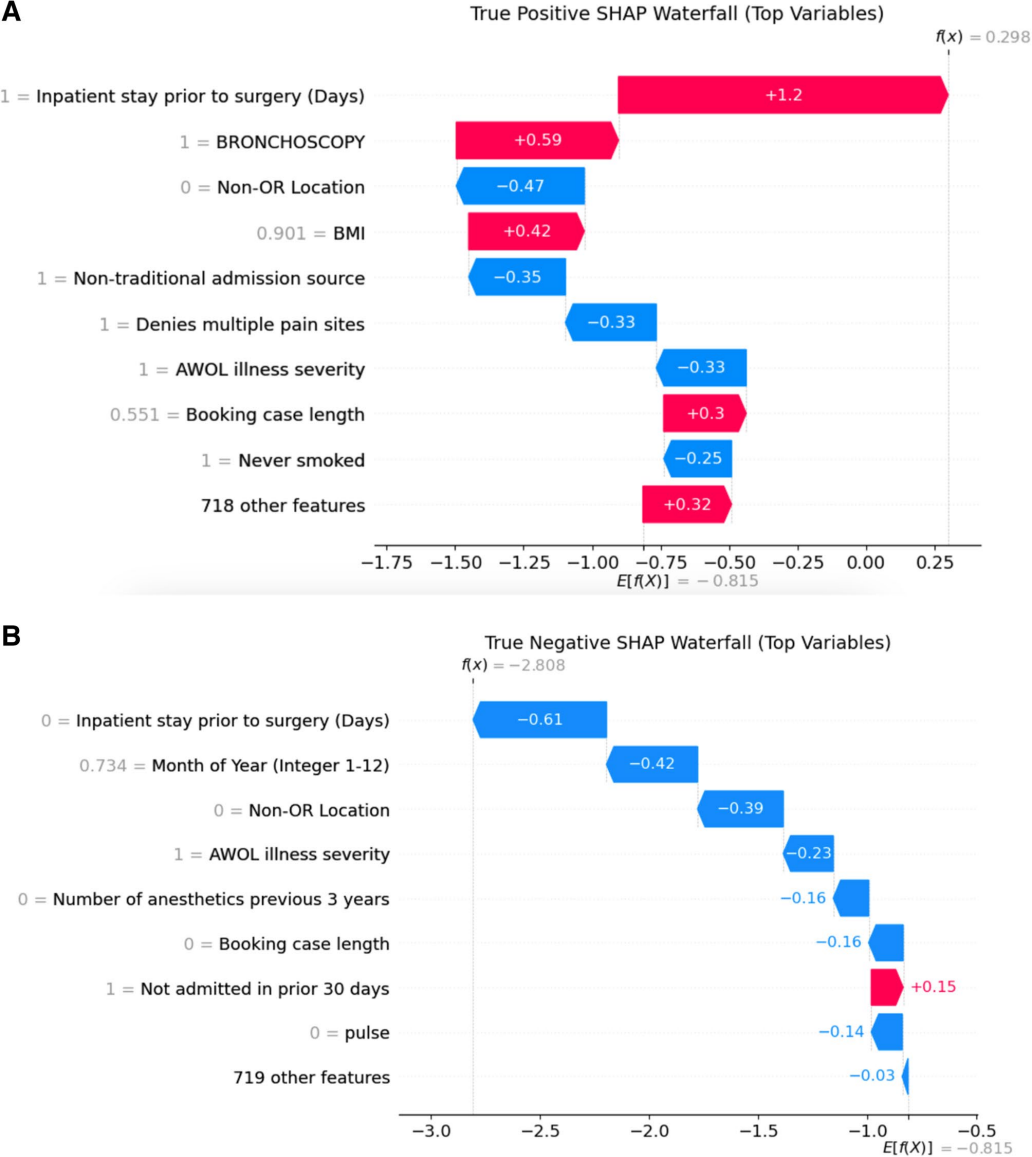
Model decision waterfall plots for two individual patient. Each bar shows a feature’s SHAP contribution—labeled with the feature and its value—and bars pointing right (red) increase risk while bars pointing left (blue) decrease risk. **A** Correctly predicted positive outcome with a high-risk patient with predominantly rightward shifts. **B** Correctly predicted negative outcome with a low-risk patient with predominately leftward shifts. (AWOL [16] = delirium‐prevention score (Age >80, WORLD backwards, Orientation, and nursing illness severity)

Individual SHAP decision (waterfall) plots are shown in Fig. 4. These plots decompose the model’s prediction for a single case into a sequence of feature contributions. Each horizontal bar represents one feature’s SHAP value—how much that feature shifted the prediction up (right, risk-increasing) or down (left, risk-decreasing)—and is labeled with the feature name and the patient’s actual standardized value (modified value compared to other patients in our cohort). Bars are sorted top-to-bottom by absolute impact, so you can follow the running cumulative score (indicated by a floating dot at the end of each bar) as successive features add or subtract from the baseline. In Fig. 4A (correctly predicted positive case), strong right-pointing bars for inpatient stay, bronchoscopy, BMI, and longer booking length drive the prediction above baseline. In Fig. 4B (correctly predicted negative case), protective features such as no inpatient stay prior to surgery and shorter booking length produce left-pointing bars that pull the prediction below baseline.

## Discussion

Using standard preoperative variables, we developed a machine learning-derived model to predict the rare outcome of an unplanned postoperative ICU admission in patients undergoing major non-cardiac surgery. Importantly, using open-source Python packages, we provide a visual explanation of the model on a global and individual scale to help corroborate clinical judgement.

The reported incidence of UIAs varies between 0.12% and 6.7% [2, 3, 6, 11]. This wide range is likely due to a difference in the pre-definition of UIAs, where some groups define ICU admissions up to 24 h postoperatively as a UIA [6, 11]. Our incidence of UIAs at 2.3% is consistent with the literature and with a stricter definition of immediate admission to the ICU from the PACU. Characteristics associated with UIAs, such as older age, higher ASA class, multiple comorbidities, and type of surgery are all consistent with prior reports [1, 6]. 

Precisely because UIAs are rare outcomes, models derived from parametric approaches often fail to provide clinically relevant predictions because they rely on normally distributed input and output variables. Similar models predicting UIAs have been developed using logistic regression; however, the models either achieved a low AUC or heavily relied on intraoperative data through the end of a surgical case [11]. Since most patients do not have a UIA, often a parametric model achieves high AUC by guessing the majority outcome without ever actually predicting the rare outcome (UIA).

XGBoost has a major advantage over other algorithms in its ability to process datasets containing missing values. In an oversimplified description, XGBoost begins with a decision tree to make positive and negative predictions, and if the output predictions from that tree are not correct, the algorithm ‘boosts’ or focuses its training on new trees to improve outcomes. This iterative learning process allows for better prediction of rare outcomes like UIAs. Our model achieved an AUC of 0.80 (95% CI 0.74–0.86) on the final test dataset.

Next, we determined two decision thresholds of 5% and 30% on the validation dataset to maximize sensitivity and specificity and increase the positive likelihood ratio of a UIA. This was a conscious decision, since the purpose of the model is to guide mobilization of perioperative resources to create an ICU bed when one is not anticipated. Prior reports have lowered the threshold for positive prediction to 5% [11]. The lower threshold maximizes sensitivity, while the higher threshold maximizes specificity. Clinically, the higher decision threshold is more relevant for proper allocation of ICU beds without depletion of resources. With the higher decision threshold, our model results in a positive likelihood ratio of 4.22, which increases the positive post-test probability of a UIA for our higher risk patients from 20% to greater than 50%. We recognize that although the absolute impact of this model still has room for improvement, the model still outperforms parametric comparison and serves as a tool for a problem with no current solution. The practical clinician may ask why a complex machine-learning method is superior to intuitive parametric methods [20]. Our results emphasize the importance of other clinically relevant metrics in evaluating the usefulness of a model, including sensitivity, specificity, and likelihood ratios, for the prediction of rare events.

We anticipate using this model in several ways. Because the model runs on preoperative data only, we can prospectively screen for unexpected ICU admissions among this patient population upon scheduling of a surgical case and allow clinicians to determine if further preoperative optimization is warranted. Second, on any given day, if the model predicts more patients needing ICU care than existing beds, the perioperative clinician can review the model’s individual decisions to help guide clinical judgement on ICU bed allocation. As hospitals continue to balance the scheduling of elective surgeries amid a pandemic, this model can be used as an extra tool for complex perioperative planning.

The open-sourced SHAP package is especially useful in understanding the decisions made by our model. Unlike classical regression models, there is no one equation that describes all the patients. Globally, the SHAP summary plot points clinicians towards the features that were important in the model’s decision making. Not surprisingly, the model found that certain types of surgeries (i.e., carotid), BMI, and certain past medical history diagnoses are highly predictive for a UIA (Fig. 3). The individual decision plots provide visual guidance that can be used proactively not only to guide perioperative planning but also to help patients with shared decision making. Furthermore, even if the model predicts incorrectly, a clinician can use expert domain knowledge to help retrain and improve future outcomes. This concept of expert-augmented machine learning will likely be important as more prediction models become available [21]. 

Bronchoscopy as a Risk Marker: in a post-hoc multivariable model, bronchoscopy had an OR of 1.77 (95% CI 0.42–7.53; *p* = 0.44) and thoracic service an OR of 2.34 (95% CI 0.57–9.60; *p* = 0.24). While not statistically significant—likely from small numbers—both estimates suggest that bronchoscopy flags patients at higher UIA risk, reflecting procedural complexity and baseline pulmonary vulnerability. We therefore recommend that bronchoscopy patients, receive enhanced preoperative pulmonary optimization (e.g., inspiratory muscle training, tailored bronchodilators, or advanced respiratory therapy).

As machine learning risk prediction tools move from retrospective validation into real-time perioperative workflows, there is an ethical imperative to incorporate their use into the informed consent process. Patients should be made aware that a predictive algorithm may influence care decisions—such as allocation of ICU resources or the intensity of preoperative optimization interventions—and understand both its benefits and limitations. Moreover, beyond bed-planning, this model holds promise as a trigger for tailored preoperative optimization (for example, targeted prehabilitation or more aggressive management of modifiable risk factors). We therefore advocate for future prospective studies to evaluate not only the tool’s predictive performance but also patient acceptance, impact on shared decision-making, and outcomes of algorithm-driven preoperative interventions.

There are several limitations to this study. First, our dataset was obtained retrospectively, and many data points that were excluded as outliers were likely due to errors in data entry. Importantly, as the hospital census is constantly in flux, there were likely patients who were intended to recover in an ICU but were temporarily admitted to the PACU due to a shortage of ICU beds. Second, although the model was trained, fine-tuned, and tested on separate datasets, the entirety of the dataset came from a single institution. We specifically chose predictor variables that are easily found across any institution; however, further validation at a separate institution is warranted. Future multicenter studies will be essential to validate, recalibrate, and generalize the model across diverse surgical populations and care environments. Third, we acknowledge that the modern anesthesiologist is approached with numerous complex clinical decision support tools, from within electronic health records to auxiliary devices [22]. Future work will need to study provider compliance and patient outcomes upon execution of the model at our institution, as well as prospective validation in real-time. Moreover, real-time, head‐to‐head comparison against experienced clinician risk assessments will be essential to determine the model’s added value in practice. Importantly, our model remains easy to implement with open source packages and has high potential for improved patient outcomes with low potential hospital cost.

## Supplementary Information

Supplementary Material 1: Supplemental Fig 1. Shows the sensitivity and specificity of two different decision thresholds for model clinical decision support. 

Supplementary Material 2: Supplemental Table 1. Variables included in model [23].

## Data Availability

The datasets used and/or analyzed during the current study are available from the corresponding author on reasonable request.

## Abbreviations

UCSF: University of California, San Francisco
ICU: Intensive care unit
UIAs: Unplanned postoperative ICU admissions
PACU: Post-anesthesia care unit
IRB: Institutional Review Board
STROBE: Strengthening the Reporting of Observational Studies in Epidemiology
TRIPOD: Transparent Reporting of a Multivariable Prediction Model for Individual Prognosis or Diagnosis
ASA: American Society of Anesthesiologists
XGBoost: eXtreme Gradient Boosting
SHAP: SHapley Additive exPlanations
ROC: Receiver operating characteristic
AUC: Area under the curve
AWOL: AWOL Score for delirium prevention [16]
