# Supplementary material for: Development of a machine learning-derived model to predict unplanned ICU admissions after major non-cardiac surgery
Source: BMC Anesthesiol. 2025 Jul 17;25:351. doi: 10.1186/s12871-025-03195-8 (PMC12273467; doi:10.1186/s12871-025-03195-8)
Supplement: Supplementary file 1 — Supplementary Material 1: Supplemental Fig 1. Shows the sensitivity and specificity of two different decision thresholds for model clinical decision support. [file 12871_2025_3195_MOESM1_ESM.docx]

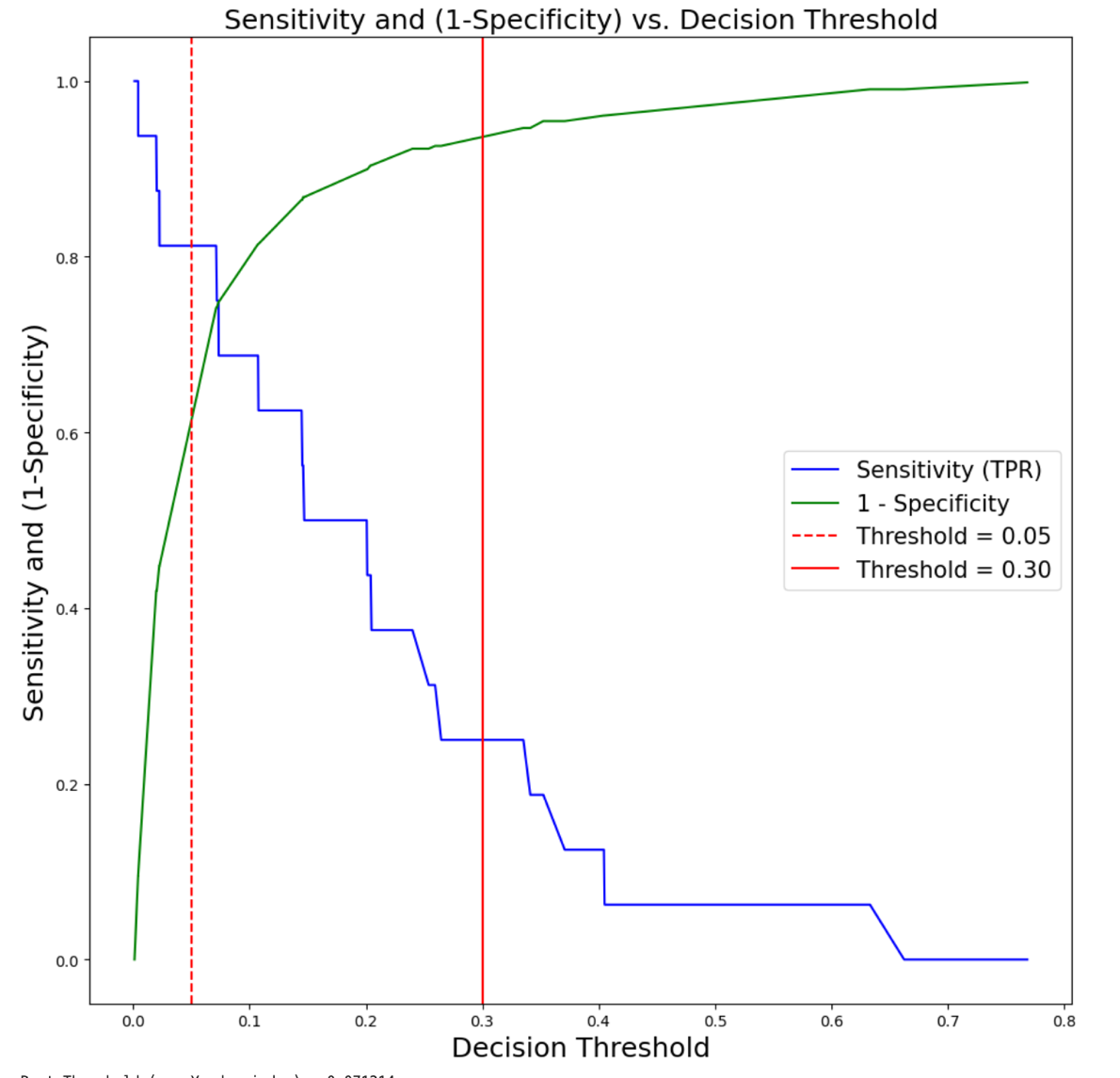


**Supplemental Fig 1**. Shows the sensitivity and specificity of two different decision thresholds for model clinical decision support.
