# Supplementary material for: Development of a machine learning-derived model to predict unplanned ICU admissions after major non-cardiac surgery
Source: BMC Anesthesiol. 2025 Jul 17;25:351. doi: 10.1186/s12871-025-03195-8 (PMC12273467; doi:10.1186/s12871-025-03195-8)
Supplement: Supplementary file 2 — Supplementary Material 2: Supplemental Table 1. Variables included in model [23]. [file 12871_2025_3195_MOESM2_ESM.docx]

Supplemental Table 1: Variables included in model

| Category Type | Original Variable |
| --- | --- |
| Numeric | month_of_year |
| Numeric | length_of_stay_day |
| Numeric | age |
| Numeric | bmi |
| Numeric | num_of_anes_enc_prev_3yrs |
| Numeric | allergies_num |
| Numeric | booking_case_length |
| Numeric | ABDOMINAL |
| Numeric | AMPUTATION |
| Numeric | ANEURYSM |
| Numeric | ANGIO |
| Numeric | AORTIC |
| Numeric | APPENDECTOMY |
| Numeric | BRONCHOSCOPY |
| Numeric | BYPASS |
| Numeric | CAROTID |
| Numeric | CHOLECYSTECTOMY |
| Numeric | COLECTOMY |
| Numeric | CYSTOSCOPY |
| Numeric | DEBRIDEMENT |
| Numeric | ECTOMY |
| Numeric | EGD |
| Numeric | ENDO |
| Numeric | EXTREMITY |
| Numeric | FISTULA |
| Numeric | FLAP |
| Numeric | FUSION |
| Numeric | GASTRIC |
| Numeric | GRAFT |
| Numeric | HEPATECTOMY |
| Numeric | HERNIA |
| Numeric | INSERTION |
| Numeric | KNEE |
| Numeric | LAPAROSCOPIC |
| Numeric | LAPAROTOMY |
| Numeric | LOWER |
| Numeric | NEPHR |
| Numeric | OPEN |
| Numeric | OSTOMY |
| Numeric | OTOMY |
| Numeric | PANCREATECTOMY |
| Numeric | PLASTY |
| Numeric | PROSTAT |
| Numeric | PUMP |
| Numeric | REMOVAL |
| Numeric | RESECTION |
| Numeric | REVISION |
| Numeric | ROBOTIC |
| Numeric | SCOPY |
| Numeric | STENT |
| Numeric | THORACIC |
| Numeric | THORACOTOMY |
| Numeric | THYROIDECTOMY |
| Numeric | TRANSPLANT |
| Numeric | TUMOR |
| Numeric | UPPER |
| Numeric | VASC |
| Numeric | VENTRAL |
| Numeric | WHIPPLE |
| Numeric | month_of_year |
| Numeric | age |
| Numeric | weightinkg |
| Numeric | bmi |
| Numeric | heightincm |
| Numeric | num_of_anes_enc_prev_3yrs |
| Numeric | allergies_num |
| Numeric | allergies_num_severe |
| Numeric | length_of_stay_day |
| Numeric | transportation_needs |
| Numeric | food_insecurity |
| Numeric | financial_resource_strain |
| Numeric | temp |
| Numeric | temp_src |
| Numeric | pulse |
| Numeric | resp |
| Numeric | spo2 |
| Numeric | glucose_manual_entry |
| Numeric | bsa_calculated_mosteller_formula |
| Numeric | awol_world_backwards^1^ |
| Numeric | awol_illness_severity^1^ |
| Numeric | awol_oriented^1^ |
| Numeric | schmid_fall_score |
| Numeric | braden_scale |
| Numeric | stratify_fall_risk |
| Numeric | problem_documented_skin_condition |
| Numeric | problem_documented_anus_and_rectum |
| Numeric | problem_documented_heent |
| Numeric | glasgow_coma_scale_score |
| Numeric | awols^23^ |
| Numeric | booking_case_length |
| Numeric | induction |
| Numeric | patient_billing_type |
| Numeric | admitting_service |
| Numeric | admission_patient_class |
| Numeric | planned_anesthesia_type |
| Numeric | ICD_A_B |
| Numeric | ICD_C |
| Numeric | ICD_D |
| Numeric | ICD_E |
| Numeric | ICD_F |
| Numeric | ICD_G |
| Numeric | ICD_H |
| Numeric | ICD_I |
| Numeric | ICD_J |
| Numeric | ICD_K |
| Numeric | ICD_L |
| Numeric | ICD_M |
| Numeric | ICD_N |
| Numeric | ICD_O |
| Numeric | ICD_P |
| Numeric | ICD_Q |
| Numeric | ICD_R |
| Numeric | ICD_S |
| Numeric | ICD_T |
| Numeric | ICD_U |
| Numeric | ICD_VWX |
| Numeric | ICD_Z |
| Categorical | day_of_surgery |
| Categorical | gender |
| Categorical | booking_codes |
| Categorical | 30_day_prior_admit |
| Categorical | 90_day_prior_admit |
| Categorical | or_service |
| Categorical | schd_day_start |
| Categorical | case_emergent |
| Categorical | date_of_service |
| Categorical | day_of_surgery |
| Categorical | gender |
| Categorical | race |
| Categorical | ethnicity |
| Categorical | allergies |
| Categorical | marital_status |
| Categorical | payor |
| Categorical | prefer_learn_new_concepts |
| Categorical | barrier_to_learning |
| Categorical | primary_language |
| Categorical | interpreter_needed |
| Categorical | smoking_status |
| Categorical | alcohol_use |
| Categorical | substance_use |
| Categorical | primary_care_physician |
| Categorical | admission_time |
| Categorical | date_of_service.1 |
| Categorical | heart_rate_source |
| Categorical | bp |
| Categorical | bp_method |
| Categorical | pain_assessment |
| Categorical | pain_level |
| Categorical | acceptable_level_of_pain |
| Categorical | pain_type |
| Categorical | pain_location |
| Categorical | pain_character |
| Categorical | effect_of_pain_on_daily_activities |
| Categorical | multiple_pain_sites |
| Categorical | pain_interventions |
| Categorical | thermoregulation_interventions |
| Categorical | respiratory_interventions |
| Categorical | access_to_transportation |
| Categorical | dressing |
| Categorical | grooming |
| Categorical | bathing |
| Categorical | in_out_bed |
| Categorical | feeding |
| Categorical | weakness_in_arms_hands |
| Categorical | weakness_in_legs |
| Categorical | bowel_bladder_habits |
| Categorical | vision_issues |
| Categorical | hearing_r_ear |
| Categorical | hearing_l_ear |
| Categorical | mobility_issue |
| Categorical | elimination |
| Categorical | history_of_falls |
| Categorical | problem_documented_neuro |
| Categorical | problem_documented_cardiac |
| Categorical | problem_documented_musculoskeletal |
| Categorical | problem_documented_genitorurinary |
| Categorical | problem_documented_psychosocial |
| Categorical | social_work_consult_needed |
| Categorical | suicide_risk |
| Categorical | pain_or_discomfort |
| Categorical | pain_relieving_factors |
| Categorical | unplanned_weight_loss |
| Categorical | difficulty_chewing |
| Categorical | difficulty_swallowing |
| Categorical | tube_feeding |
| Categorical | total_parental_nutrition |
| Categorical | presence_of_pressure_ulcer |
| Categorical | nonhealing_wound |
| Categorical | dietician_consult_needed |
| Categorical | o2_device |
| Categorical | braces_devices_sensory_aids |
| Categorical | sleep_habit_details |
| Categorical | arrived_from |
| Categorical | awol^1^ |
| Categorical | primary_anesthesiologist |
| Categorical | primary_surgeon |
| Categorical | case_location |
| Categorical | anes_staff_on_case |
| Categorical | surgery_staff |
| Categorical | booking_codes |
| Categorical | procedure_list |
| Categorical | 30_day_prior_admit |
| Categorical | 90_day_prior_admit |
| Categorical | patient_class |
| Categorical | patient_admission_source |
| Categorical | or_service |
| Categorical | emergency_contact_listed |
| Categorical | case_classification |
| Embedding | past_medical_history |
